# Preoperative nutritional evaluation of prostate cancer patients undergoing laparoscopic radical prostatectomy

**DOI:** 10.1371/journal.pone.0262630

**Published:** 2022-02-02

**Authors:** Wang Shu, Wu Tao, Hu Chunyan, Fan Jie, Liu Yuan, Xu Yan, Zhang Huan, Xie Liang

**Affiliations:** 1 Department of Urology Surgery, Affiliated Hospital of North Sichuan Medical College, Nanchong, China; 2 Department of Digestive System, Affiliated Hospital of North Sichuan Medical College, Nanchong, China; 3 Health Management Center, Affiliated Hospital of North Sichuan Medical College, Nanchong, China; 4 Department of Hepatobiliary Surgery (2), Affiliated Hospital of North Sichuan Medical College, Nanchong, China; Stanford University School of Medicine, UNITED STATES

## Abstract

**Background and objective:**

Prostate cancer (PCa) is one of the most common malignant tumors in men. Geriatric Nutritional Risk Index (GNRI) is an objective index for evaluating nutritional status of elderly people over 65 years old. The aim of the current study was to explore the correlation and predictive value between GNRI and postoperative recovery and complications in PCa patients undergoing laparoscopic radical prostatectomy (LRP).

**Methods:**

Taking 98 as the GNRI boundary value, 96 PCa patients (aged≥65 y) undergoing LRP in the Department of Urology, Affiliated Hospital of North Sichuan Medical College from January 2018 to December 2020 were grouped into malnutrition group (MNg, 34 patients, 35.4%) and normal nutrition group (NNg, 62 patients, 64.6%). Basic information, laboratory examination indexes, operation conditions, postoperative complications and postoperative recovery indexes of patients were recorded and retrospectively analyzed. Clavien-Dindo Classification System (CDCS) was used to assess postoperative complications. T-test was used to analyze differences between the two groups. ROC curve was generated to determine the predictive value of GNRI for postoperative complications.

**Results:**

Percentage of complications was significantly higher in MNg group compared with that in NNg group (*P* < 0.01). The average grade based on CDCS was significantly lower in NNg group compared with that in MNg group (*P* < 0.01). Body weight, Body Mass Index (BMI), preoperative hemoglobin value (HGB), serum albumin (ALB) values of MNg and NNg were significantly positively correlated with GNRI (P<0.01). Incidence and severity of postoperative complications of MNg patients were significantly higher compared with those of NNg patients (P<0.05). Average hospitalization cost of MNg patients was higher in MNg patients compared with that of NNg patients (P<0.05). Duration of post-anesthesia care unit (PACU), duration of antibiotic use and duration of indwelling drainage tube were longer in MNg patients compared with those in NNg patients (P<0.05). Furthermore, volume of indwelling drainage tube was higher in MNg patients compared with that in NNg patients (P<0.05).

**Conclusion:**

GNRI is an effective and reliable tool for evaluation of preoperative nutritional status of prostate cancer patients. The findings showed that GNRI is correlated with postoperative recovery and complications, and is an effective predictive marker.

## Introduction

Prostate cancer (PCa) is one of the most common malignant tumors in men [1]. Pca is the second-leading male malignant tumor with an incidence of 2.93/million worldwide [2]. Incidence of PCa is correlated with age, and is higher in men with urogenital system diseases [3]. The European Urological Association recommends surgery as the conventional treatment for PCa. Laparoscopic radical prostatectomy (LRP) is the standard treatment for limited PCa, and is recommended as the first-line treatment by most guidelines [4, 5]. However, laparoscopic surgery in urology is associated with postoperative complications, and incidence of complications increases with challenges in performing surgery.

Nutrition is the basis of maintaining normal physiological function of the human body. The nutritional status of patients is closely related to prognosis of surgical operation [6]. Older patients (aged ≥ 65) have higher incidence of complications and mortality owing to advanced age and effects of the cancer itself [7]. Previous studies report that nutritional factors are correlated with occurrence and progression of PCa [8].

Geriatric Nutritional Risk Index (GNRI) is an objective index for evaluating nutritional status of elderly people over 65 years old. It has been used effectively in predicting prognosis of patients with lung cancer, esophageal cancer, soft tissue sarcoma, renal cancer and pancreatic cancer [9–13]. However, currently no clinical study has explored use of GNRI in predicting complications and postoperative recovery of PCa patients undergoing LRP. In order to fill this gap, the aim of the current study was set to explore the correlation and predictive value between GNRI and postoperative recovery and complications in PCa patients undergoing LRP.

## Materials and methods

### Patients

A total of 96 PCa patients undergoing LRP in the Department of Urology of Affiliated Hospital of North Sichuan Medical College between June 2016 to June 2021 were enrolled in this study. All patients were Han males. Inclusion criteria were as follows: 1. Prostate biopsy performed in our hospital before operation, and PCa confirmed by pathological diagnosis; 2. Age ≥ 65 years; 3. patients who only underwent LRP during hospitalization. Exclusion criteria: 1. Patients with malnutrition caused by other severe diseases, 2. Patients with urinary calculi, other tumors and other urinary diseases, 3. Patients with infectious diseases (such as respiratory tract infection and pulmonary infection) or other chronic wasting diseases.

### Methods

#### Research indicators

Height, weight, age, hospitalization costs, postoperative length of stay and other basic information of patients were recorded. Moreover, data on operation duration, operation method, intraoperative blood loss, duration of post-anesthesia care unit (PACU), duration of postoperative catheter indwelling, duration of indwelling drainage tube, volume of indwelling drainage tube, duration of antibiotic use were obtained from medical records. In addition, data on complications such as ileus, urethrorectal fistula, ureteral injury, cardio cerebral vascular accident, postoperative infection, postoperative massive hemorrhage, urinary retention, urinary incontinence were obtained from medical records. Blood routine and liver function tests were performed to determine hemoglobin value (HGB), total lymphocyte (TLC) count, white blood cell (WBC) count and serum albumin (ALB) level before and after the operation.

#### Evaluation and grading standard of complications

Clavien Dindo classification system (CDCS) was used for grading of complications [14]. Postoperative complications were classified as grade I-V based on the CDCS grading system. Grade I comprised any postoperative abnormalities that do not require drug treatment, surgery, endoscopy or intervention. Grade I cases were treated using antiemetics, antipyretics, analgesics, diuretics, electrolytes and physical therapy. For grade II, in addition to the drugs used for grade I complications, blood transfusion and total parenteral nutrition were incorporated. Surgical, endoscopic or interventional intervention were used for grade III. For grade Ⅲa no intervention was administered under general anesthesia Whereas for Grade Ⅲb intervention under general anesthesia was required. Grade IV included life-threatening complications requiring ICU treatment or intermediate care. Grade IVa comprised single organ dysfunction (including dialysis treatment). Grade IVb comprised multiple organ dysfunction. Grade V included cases of death. In cases of multiple complications on one patient, only the most severe complication was recorded.

Urinary incontinence was evaluated based on the International Consultation on Incontinence Questionnaire—Short Form (ICIQ-SF) [15], which was filled through telephone follow-up. Postoperative infections recorded included urinary tract infection, pulmonary infection, abdominal and retroperitoneal infection. Incision complications included hematoma, fat liquefaction, incision dehiscence or infection.

#### Nutritional status indicator

Nutritional status was evaluated based on the geriatric nutritional risk index (GNRI) and Body Mass Index (BMI). Height, weight and ALB of the patients were determined routinely one week before operation. In the current study, GNRI and BMI was calculated based on data obtained from medical records. GNRI was calculated using the formula: GNRI = 1.489 × ALB (g/L) + 41.7 × (actual weight / ideal weight). Male ideal weight was calculated as height (cm) - 100 - [(height (cm) - 150) / 4]. The ratio was 1 for cases where the preoperative actual weight ≥ the ideal weight [16]. If the patient’s GNRI ≤ 98, he/she will be grouped into malnutrition group (MNg); On the contrary, he/she will be grouped into normal nutrition group (NNg) [17]. BMI was calculated by taking a person’s weight, in kilograms, divided by their height, in meters squared, or BMI (kg/m^2^) = weight (kg)/ height (m) ^2^ [18].

#### Ethical statement

This study was approved by the medical ethics committee of Affiliated Hospital of North Sichuan Medical College, Nanchong, China. Informed verbal consents were obtained from all patients as telephone follow-up communication were conducted through patients or their families. Verbal consent was documented in the form of a written record and approved by the medical ethics committee of our hospital. The study was conducted following the guidelines outlined in the declaration of Helsinki.

#### Statistical analyses

All data were entered and analyzed using *SPSS* 22.0 (SPSS Inc., Chicago, IL, USA), *GraphPad Prism* 8 (GraphPad Software, LaJolla, CA, USA) or *MedCalc* 20.0 software (MedCalc Software, Mariakerke, Belgium). Mean ± standard deviation was used to describe continuous data that followed normal distribution. T-test was used to explore differenced in nutritional indexes of patients, general characteristics of patients, perioperative indexes and hematological indexes between the two groups. A ROC curve was generated to analyze the predictive factor, and the area under the ROC curve (AUC) was calculated.

## Results

### General characteristics of research subjects

A total of 96 patients, aged 65–83 years, with an average age of 72.50 ± 4.82 years were included in this study. Height of the patients ranged between 150 and 178cm, with an average height of 164.24 ± 6.10cm, their weight ranged between 44 and 90kg, with an average height of 62.90 ± 9.08kg. BMI (kg/m^2^) of the patients ranged between 17.2–31.1, with an average BMI of 23.32 ± 2.88 and a GNRI score ranging between 84.2–118.0, with an average GNRI score of 101.22 ± 6.30.

Out of the 96 patients, 34(35.4%) Patients were grouped into MNg and 62(64.6%) patients were grouped into NNg. Age, height, weight, BMI, HGB, ALB, PSA, intraoperative blood loss and intraoperative blood transfusion were compared between the two groups as shown in Table 1. Analysis showed that weight, BMI, preoperative HGB and ALB values were significantly different between the two groups (P < 0.01). Notably, no significant differences were observed in age, height, PSA, intraoperative blood loss and intraoperative blood transfusion between the two groups (P > 0.05).

**Table 1:** General characteristics, preoperative laboratory indexes and intraoperative conditions of patients.

Indexes	Total (N = 96)	NNg (N = 62)	MNg (N = 34)	NNg vs MNg	
				*t*	*P*
**Age (years)**	72.50 ± 4.82	71.90±4.38	73.59±5.43	1.653	0.102
**Height (cm)**	164.24 ± 6.10	164.60±5.74	163.59±6.77	-0.773	0.442
**Weight (kg)**	62.90 ± 9.08	65.37±8.35	58.38±8.72	-3.863	<0.001^b^
**BMI (kg/m** ^**2**^ **)**	23.32 ± 2.88	24.15±2.80	21.80±2.41	-4.121	<0.001^b^
**HGB (g/L)**	133.12 ± 13.84	138.03±11.78	124.15±12.91	-5.339	<0.001^b^
**ALB (g/L)**	40.97 ± 3.78	42.77±2.64	37.70±3.34	-7.641	<0.001^b^
**PSA (ug/L)**	22.50 ± 21.82	21.58±19.76	24.18±25.39	0.555	0.580
**Intraoperative blood loss (ml)**	345.10 ± 268.99	312.58±236.66	404.41±314.88	1.613	0.110
**Intraoperative blood transfusion (ml)**	57.81 ± 175.07	37.90±138.10	94.12±225.55	1.515	0.133

^a^ Correlation is significant at 0.05 level (2-tailed).

^b^ Correlation is significant at 0.01 level (2-tailed).

### Comparison and predictive value of GNRI in postoperative complications between NNg and MNg groups

Incidence of urinary incontinence, infection and incision complications in MNg was significantly higher the MNg group compared with those in NNg group (*P* < 0.05, Table 2). Notably, urethrorectal fistula, ureteral injury, and cardio cerebrovascular accident were only observed in MNg group. Hemorrhage level was higher in MNg group compared with the level in NNg group. However, the difference was not statistically significant owing to the limited sample size. Average incidence of complications (person-time) was significantly higher in MNg group compared with the incidence in NNg group (t = 4.033, *P* < 0. 01).

**Table 2:** Comparison of postoperative complications between the two groups.

Complications	NNg (N = 62)	MNg (N = 34)	χ^2^	*P*
**Urinary incontinence**	7(11.3%)	16(47.1%)	15.420	<0.001^b^
**Urethrorectal fistula**	0(0%)	2(5.9%)	3.686	0.055
**Ureteral injury**	0(0%)	1(2.9%)	1.824	0.177
**Cardiovascular and cerebrovascular accident**	0(0%)	2(5.9%)	3.686	0.055
**Incomplete ileus**	4(6.5%)	2(5.9%)	0.012	0.913
**Infection**	21(33.9%)	19(55.9%)	4.377	0.036^a^
**Massive hemorrhage**	1(1.6%)	2(5.9%)	1.308	0.253
**Incision complications**	5(8.1%)	8(23.5%)	4.486	0.034^a^
**Total** (Average person-time)	38(0.613)	52(1.529)	4.033	<0.001^b^

The findings showed that complication free percentage was significantly higher in NNg group compared with that of MNg group (Table 3). Incidence of grade I, II and III complications were lower in NNg group compared with that of MNg group (**χ**^**2**^ = 12.500, *P* < 0.01). Notably, no grade IV and V complications were observed in both groups. Percentage of complications was significantly higher in MNg group compared with that in NNg group (**χ**^**2**^ = 9.623, *P* < 0.01). The average grade based on CDCS was significantly lower in NNg group (0.58 ± 0.69) compared with that in MNg group (1.21 ± 0.91) (t = 3.774, *P* < 0.01).

**Table 3:** Comparison of CDCS between the two groups.

CDCS	NNg (N = 62)	MNg (N = 34)	χ^2^	*P*
**0 (no complication)**	33(53.2%)	7(20.6%)	12.500	0.001 ^b^
**I**	22(35.5%)	17(50.0%)		
**II**	7(11.3%)	6(17.6%)		
**III**	0(0%)	4(11.8%)		
**Ⅳ**	0(0%)	0(0%)		
**V**	0(0%)	0(0%)		
**I-V Total**	29(46.8%)	27(79.4%)	9.623	0.002 ^b^

**Fig 1:**
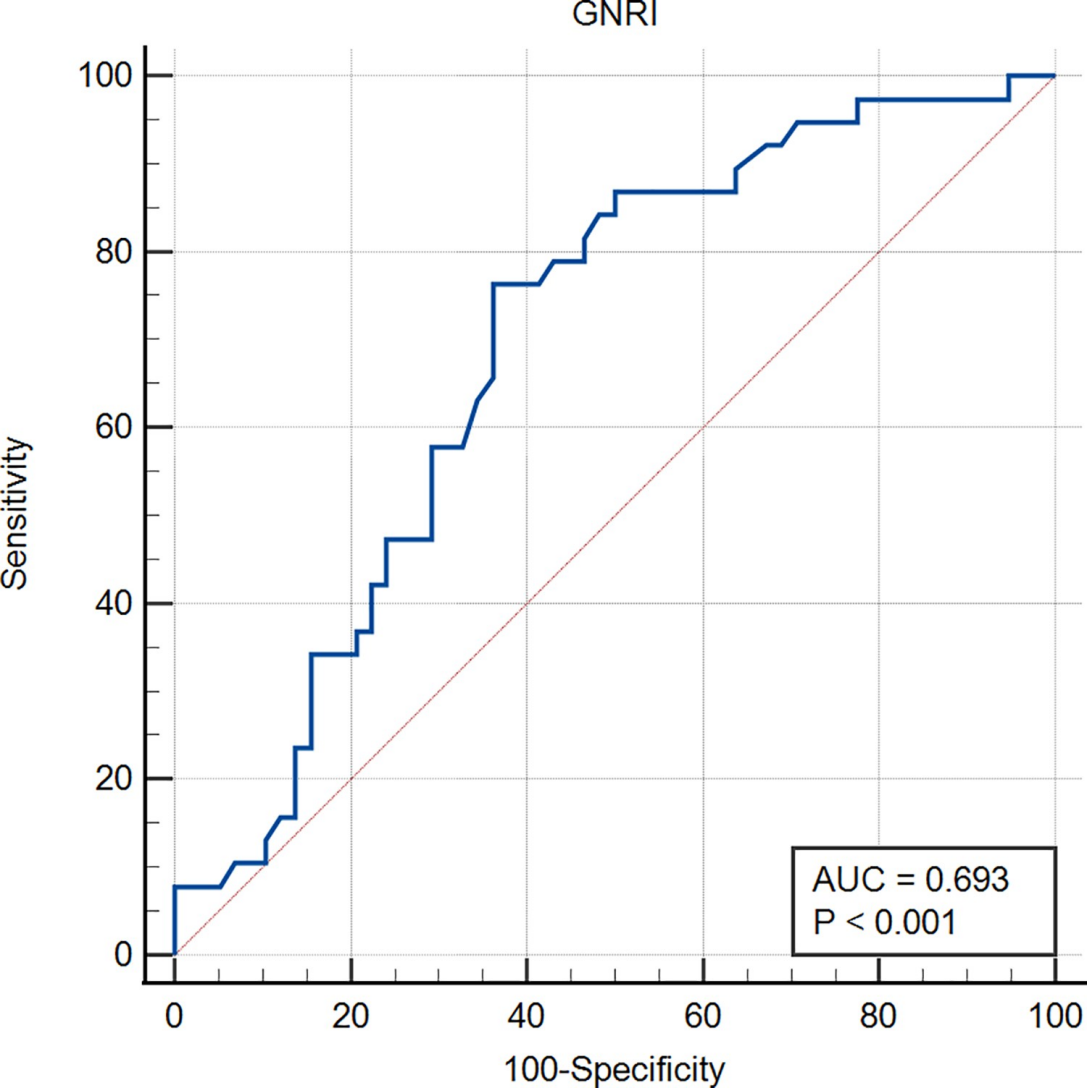
ROC curve of GNRI for prediction of postoperative complications.

Further, the 96 patients were divided into non-complication group and complication group based on occurrence of complications. ROC curve was used to determine the predictive value of GNRI for postoperative complications. The area under the curve (AUC) was 0.693 (95% CI = 0.590–0.783, Fig 1). The findings showed a GNRI cutoff value of 100.7, sensitivity of 0.763, specificity of 0.638, and highest Youden index of 0.401.

### Comparison of postoperative recovery between NMg and MNg

**Fig 2:**
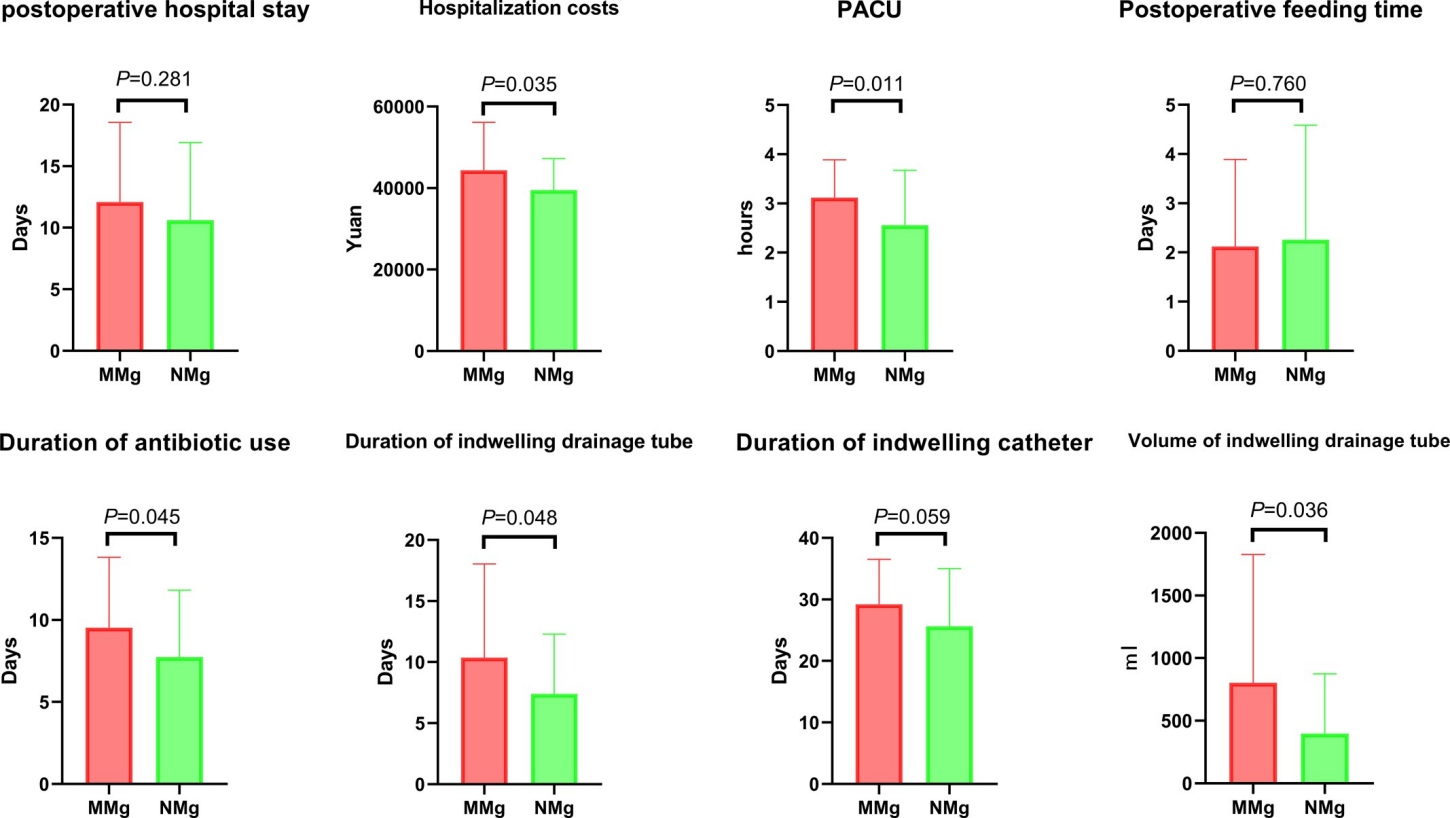
Comparison of postoperative recovery characteristics between the two groups.

Average postoperative hospital stay-period of patients in MNg and NNg groups were 12.09 ± 6.48 and 10.61 ± 6.31 days, respectively (Fig 2A). The findings showed no significant difference in time of hospital stay between the two groups (*P* > 0.05). Average hospitalization costs of MNg and NNg patients were 44358.65 ± 11767.69 and 39505.33 ± 7721.26 yuan, respectively (Fig 2B). Statistical analysis showed that the average hospitalization costs were significantly higher in MNg patients compared with those for NNg patients (*P* < 0.01). Average duration of PACU of MNg and NNg patients were 3.12 ± 0.77 and 2.56 ± 1.12 hours, respectively (Fig 2C). Analysis showed that the average duration of PACU was longer in MNg patients compared with that of NNg patients (*P* < 0.05). The findings showed that the average postoperative feeding time of MNg and NNg patients was 2.12 ± 1.77 and 2.26 ± 2.33 days, respectively (Fig 2D). Analysis showed no statistical difference in postoperative feeding time between the two groups (*P* > 0.05).

Average duration of antibiotic use in MNg and NNg patients was 9.53 ± 4.29 and 7.73 ± 4.09 days, respectively (Fig 2E). Analysis showed that the average duration of antibiotic use was longer in MNg group compared with that of NNg patients (*P* < 0.05). Average duration of indwelling drainage tube in MNg and NNg patients was 10.35 ± 7.69 and 7.39 ± 4.90 days, respectively (Fig 2F). Analysis showed that the average duration of indwelling drainage tube was longer in MNg patients compared with that of NNg patients (*P* < 0.05). Average duration of indwelling catheter in MNg and NNg patients was 29.21 ± 7.30 and 25.65 ± 9.38 days, respectively (Fig 2G). Analysis showed no statistical difference in average duration of indwelling catheter between the two groups (*P* > 0.05). Average volume of indwelling drainage tube in MNg and NNg patients was 802.06 ± 1025.82 and 397.74 ± 475.67 ml, respectively (Fig 2H). Analysis showed that the average drainage volume was higher in MNg patients compared with that of NNg patients (*P* < 0.05).

## Discussion

### Selection of nutritional indicators

More than 50 different nutrition assessment tools have been developed in previous studies [19]. Most of these tools have been used to predict the prognosis of urological patients. Li et al. reported that elevated pretreatment prognostic nutritional index (PNI) is an effective prognostic indicator for PCa patients treated with androgen deprivation therapy [20]. Analysis using Nutritional Risk Screening (NRS-2002) tool showed that 21% and 55% of patients were at risk of malnutrition before radical cystectomy [21]. However, analysis using Patient-Generated Subjective Global Assessment (PG-SGA) showed that 28% of patients were moderately or severely malnourished before surgery [22]. Arshad et al. reported that Mini Nutritional Assessment (MNA) is correlated with serum albumin levels, and can be used for evaluation of malnutrition in end-stage renal disease [23]. In addition, GNRI is widely used in nutritional evaluation of urinary tumors such as renal cell carcinoma and prostate cancer [24–27].

GNRI is an effective and reliable tool for nutrition assessment [19]. GNRI was established by Bouillanne et al. in 2005 and is based on ALB level and the ratio of actual body weight to ideal body weight [16]. GNRI is a suitable tool for evaluating nutrition status in elderly community patients and inpatients over 65 years old. A low GNRI score is associated with severe malnutrition. GNRI has a high sensitivity, good specificity and low false positive rate [28]. In addition, it is a simple and easy to use screening method with high operability. GNRI is used to predict prognosis and postoperative complications of several malignant tumors. However, level of BMI or serum albumin is not associated with prognosis of cancer patients [17, 29].

Incidence and mortality of PCa increases with age. A previous study reported that more than 70% of PCa patients in the world were over 64 years old in 2017. In addition, 80% of cases of PCa deaths in 2017 were of patients more than 65 years old [30], which is consistent with the applicable age range of GNRI.

The findings of the current study showed no significant differences in age, height, PSA, intraoperative blood loss, intraoperative blood transfusion and other baseline indicators between the two groups. Preoperative nutritional indicators such as weight, BMI, HGB, ALB of PCa patients were significantly positively correlated with GNRI. This finding indicates that GNRI indicates the nutritional status of patients, and is not interfered by other factors. Therefore, GNRI was selected for prediction of postoperative recovery and complications of PCa patients undergoing LRP.

### Relationship between nutritional status and postoperative complications in PCa patients

Average CDCS grade was significantly higher in MNg group compared with that of NNg group. Notably, the findings showed no grade III complications in MNg patients. In addition, no grade IV and V complications were observed in the two groups which can be attributed to the small sample sizes. Zhou J et al. reported that malnutrition is associated with postoperative complications and higher CDCS grade [31]. In patients with malnutrition, the body is in a state of nutritional risk, and several compensatory changes occur in body organs and tissues to adapt to this state. These changes include decreased muscle strength, prolonged recovery time of the whole body and wounds, decreased immunity, delayed wound healing, weakened function of neutrophils, macrophages and lymphocytes [32]. These changes result in severe postoperative complications in patients with malnutrition.

Incidence of complications in MNg patients was higher compared with that of NNg patients. In addition, incidence of urinary incontinence, infection and incision complications were significantly higher in MNg patients compared with the incidence in NNg patients.

Maintenance of urinary continence in men is mainly dependent on bladder function and urethral sphincter system. The main approach for reducing urinary incontinence is through dissection of prostate and its surrounding tissues carefully during operation, protecting the distal sphincter system and its innervating nerve and supporting tissue [33]. However, other related factors including age and physical condition of the patients are associated with urinary continence. Wiltz et al. [34] and Kadono et al. [35] reported that BMI >30 kg/m^2^ was an independent predictor of worse continence outcomes of patients who underwent prostatectomy after a 12-month follow-up. The findings of the current study showed that incidence of urinary incontinence in malnutrition patients was higher compared with that in normal nutrition patients. Elderly patients in China (mainly in rural areas) are more emaciated compared with those in Europe and the United States. Excessive emaciation leads to less activity similar to obesity, more time in bed, and it may induce hypoglycemia and results in reduced exercise [36], thus increasing incidence of urinary incontinence. In addition, dysfunction of urethral sphincter and autonomic contraction of pelvic floor muscle caused by malnutrition may be associated with urinary incontinence.

Postoperative infection is the most common complication of LRP. Studies report that malnutrition can lead to fluid overload and accumulation of inflammatory markers such as Interleukin 6 and tumor necrosis factor-alfa, thus aggravating postoperative infections [37, 38]. The findings of the current study showed that incidence of postoperative infection was higher in the MNg group compared with that of NNg patients. Preoperative HGB and ALB levels were lower in MNg patients compared with those of NNg patients. Furthermore, immunity duration of MNg patients was lower compared with that of NNg patients, implying that MNg patients have a higher risk of infection. Moreover, average duration of indwelling drainage tube and indwelling catheter in MNg group was longer compared with that in NNg group. This implies that the normal physiological structure of urethra was destroyed, thus reducing the function of intestinal mucosa prevent bacterial infections. Moreover, long-term indwelling catheter increases bacterial retrograde access through the catheter, resulting in urinary tract infections [39]. Increase in abdominal and pelvic exudates caused by hypoalbuminemia [40] and long-term indwelling of drainage tube [41] are two important risk factors leading to increased risk of abdominal infection in the MNg group.

Tang et al. reported that hypoalbuminemia caused by malnutrition directly leads to secondary pulmonary infections [42]. Malnutrition induces emaciation and weakness thus patients have to stay in bed for a long time after operation. As a result, body fluid overload leads to pulmonary edema and congestion [43], and neutropenia caused by decreased immunity [44], thus increasing risk of postoperative pulmonary infection.

Low preoperative ALB results in defective body enzyme production ability, poor ability of tissue and organ self-repair, resulting in delayed wound healing. Notably, hypoalbuminemia significantly affects humoral immunity, which can cause pathogen translocation, conditional pathogen transformation, and fungal reproduction [45, 46]. In addition, blood supply under the incision is reduced due to emaciation, thus increasing risk of infection on the incision area, tissue necrosis, and dehiscence [47].

In the current study, the best Youden value, sensitivity and specificity was obtained with GNRI = 100.7 as the cut-off value. This value was used for prediction of occurrence of postoperative complications. The findings showed high sensitivity and an acceptable specificity in prediction of postoperative complications. In line with the principle of operation safety first and adequate preoperative preparation, nutritional support should be ensured before operation to improve nutritional status of patients. The sample size of this single center study was small; therefore, the ROC curve was not smooth enough, and only a preliminary cut-off value was obtained. Further multi-center studies with large sample size should be conducted to obtain a more accurate cut-off value, to guide on preoperative nutritional status evaluation of PCa patients undergoing LRP.

### Relationship between nutritional status and postoperative recovery in patients with PCa

The findings showed that duration of PACU in MNg group was longer compared with that of NNg patients which was consistent with findings from previous studies [48]. Decreased ALB level and lower body weight in MNg patients can prolong metabolism time of sevoflurane and other anesthetics [49], resulting in slower anesthesia recovery of patients. Therefore, anesthesiologists have to observe patients for a longer time, due to the longer duration of PACU in MNg patients.

Findings showed that ALB level was lower in MNg group compared with that in NNg patients, thus may result in more intra-abdominal fluid leakage, higher infection and incision complications. In addition, lower ALB level may lead to more release of more inflammatory exudate, thus increasing duration of indwelling drainage tube and volume of indwelling drainage tube. Higher incidence of infection complications and incision complications of MNg patients, and complications of urethrorectal fistula, ureteral injury and massive hemorrhage, increase postoperative recovery time of MNg patients thus resulting in prolonged use antibiotics.

Notably, the findings of the current study showed no significant difference in WBC values between the two groups on the 3rd postoperative day. However, WBC values in MNg group on the 7th postoperative day were significantly higher compared with those in NNg patients on the 7th postoperative day (*P* < 0.05). These changes may result in increased duration of antibiotic use in MNg patients. A large number of previous studies have shown that malnutrition does increase the risk of infection, forcing patients to use antibiotics for a longer time, and even increasing the risk of death [50–53].

Low levels of ALB and HGB of MNg result in significant increase in use of enteral and parenteral nutrition preparations, albumin and blood products during hospitalization. In addition, low immunity of MNg and a series of infection related complications results in use of stronger antibiotics. Longer duration of PACU treatment significantly increases hospitalization costs of MNg compared with those for NNg patients, thus increasing patient burden. The results of this study are consistent with the past. Malnutrition may aggravate the burden of hospitalization in varying degrees [50, 54–56].

The current study had a few limitations. The sample size used in the study was small. In addition, it was a retrospective study therefore it may have survey bias and lack some clinical data, such as the smoking situation and cancer grades. Furthermore, biases may be caused by difference in expertise of different surgeons that performed the surgery. Therefore, further prospective studies with larger samples size should be conducted to validate the findings of the current study. Additional internal or external cross-validation results may provide evidence of reliability of GNRI.

## Conclusion

**In summary, GNRI is an effective and reliable tool for evaluating preoperative nutritional status of PCa. GNRI is correlated with postoperative recovery and complications.** Preoperative GNRI examination and effective preoperative nutritional support is important for PCa patients undergoing LRP.

## Supporting information

S1 Table Data (English). (XLSX)

S1 File PLOSOne clinical studies checklist. (DOCX)

S2 File STROBE checklist v4 combined PlosMedicine. (DOCX)
